# Toxicological effects of a difenoconazole fungicide on a non-target butterfly

**DOI:** 10.1007/s10646-026-03133-5

**Published:** 2026-07-31

**Authors:** Klaus Fischer, Jawaria Khalid, Mine Yilmazer, Ange Raharivololoniaina

**Affiliations:** Department of Biology, Institute for Integrated Natural Sciences, University of Koblenz, Universitätsstraße 1, D-56070 Koblenz, Germany

**Keywords:** Biodiversity loss, Crop yield, Ecotoxicology, *Pieris napi*, Sub-lethal effects, Triazole fungicide

## Abstract

Agricultural intensification, driven by increasing food demand, is one of the main drivers of global biodiversity loss. Pesticides, including fungicides, may increase crop yield but pose substantial threats to the environment. However, our current knowledge on the effects of fungicides on biodiversity suffers from less research interest compared to other pesticides and a neglect of insect herbivores. We here investigate the effects of a commercially available, ready-to-use difenoconazole fungicide on a non-target insect, the butterfly *Pieris napi*, under controlled laboratory conditions. When being fed with fungicide-treated host plants, butterfly larvae showed a strongly reduced survival (15%) compared to controls (60%). Survival in larvae treated with the fungicide plus an insecticide was only 1%. Sublethal effects of the fungicide included prolonged development and reduced adult size. Our results indicate a surprisingly high toxicity of the tested fungicide formulation on *P. napi*. Based on the available literature, we speculate that triazole fungicides may be in general toxic to Lepidoptera and potentially other herbivorous insects. This study contributes to the growing evidence that fungicides may well be involved in the loss of insect biodiversity in agricultural landscapes at the global scale.

**Supplementary Information:**

The online version contains supplementary material available at https://doi.org/10.1007/s10646-026-03133-5.

## Introduction

The human population has increased eightfold within the last two centuries; it has reached 8.2 billion in 2024 and is expected to reach 10 billion at the end of the 21st century (Pison and Poniakina 2024). This rapid increase exerts strong pressure on land use and food demand. Increases in the global crop demand by 100–110% from 2005 to 2050 (Tilman et al. 2011) and in food demand by 35–56% from 2010 to 2050 (van Dijk et al. 2021) have been forecasted. The increasing food demand is thus an important driver of land use change and agricultural intensification, having a large environmental impact (Fusco et al. 2023; Gallardo 2024; Godfray et al. 2024). For instance, agricultural intensification, including growing monocultures of high-yielding crops and increased applications of fertilizers and pesticides, is an important driver of biodiversity loss (Raven and Wagner 2021; Abudulai et al. 2022; Mancini et al. 2023).

The use of agrochemicals may in the short-term increase crop yield and food security by reducing the impacts of pests and plant diseases (Paramesh et al. 2023; Zhang et al. 2024; Perobelli 2025), while carrying substantial long-term risks (see below). Accordingly, the application of chemical pesticides has strongly increased over last decades, with rates surpassing that of other global change drivers including greenhouse gas emissions (Bernhardt et al. 2017; Shattuck et al. 2023; Wolfram et al. 2026). Currently, about 3.5 million tonnes of pesticides are applied globally per year (FAO 2024). Regardless of some positive effects, pesticides pose substantial threats to human health, water quality, and biodiversity (Tang et al. 2021; Zhou et al. 2025). Despite these risks, research devoted to understanding the impacts of pesticides remains considerably limited compared to other global change drivers (Bernhardt et al. 2017), likely due to the complexity of studying numerous substances, concentrations, and species, which makes such investigations both time-consuming and costly.

The extensive use of pesticides has been suggested to be an important driver of global biodiversity decline by reducing survival, reproduction, and growth of a variety of organisms ranging from microorganisms and plants to invertebrates and vertebrates (e.g., Geiger et al. 2010; McMahon et al. 2012; Brühl et al. 2021; Wan et al. 2025). Thus, pesticides may have substantial impacts on non-target organisms, but such effects are still poorly understood, especially in (herbivorous) insects (Sedlmeier et al. 2025; Wan et al. 2025). Moreover, most interest in the given context has focused on effects of insecticides on beneficial insects such as pollinators, with a special emphasis on bees (e.g., Stanley et al. 2015; Dirilgen et al. 2023; Jütte et al. 2023; Nicholson et al. 2024). Overall, the vast majority of ecotoxicological studies to date involve either aquatic invertebrates or beneficial terrestrial insects such as (bee) pollinators or predators (Sedlmeier et al. 2025). In contrast, herbivorous insects, accounting for ca. 50% of all insect species, have been largely neglected (Pisa et al. 2015; Riihimäki et al. 2025; Sedlmeier et al. 2025). This applies also to the current EU insecticide registration protocol, which does not include this vast insect group (Sedlmeier et al. 2025). Another shortcoming in our current knowledge on the effects of pesticides on non-target organisms is that fungicides have generally received less attention compared to herbicides and insecticides (Zubrod et al. 2019; Olaya-Arenas et al. 2020). This may be related to the wide-held notion that fungicides are less harmful to non-fungal biodiversity than other pesticides, although toxicological effects on non-target organisms have been repeatedly shown (e.g., Bozdogan 2014; Marinho et al. 2020; Gomes et al. 2021; Lebrun et al. 2021; Jorge-Escudero et al. 2022; Wan et al. 2025).

Here, we focus on the effects of a commercially available fungicide (“Compo Duaxo^®^”), that uses difenoconazole as active ingredient, on the butterfly *Pieris napi*. Difenoconazole is a typical triazole fungicide with high efficiency and a broad spectrum, which is widely used in agriculture (Qin et al. 2023). In addition, we include an insecticide (“Spruzit^®^ Pest-Free Rose Spray”) plus fungicide treatment as a negative control. Butterflies are important pollinators with high sensitivity to environmental change, making them highly suitable models for our purpose (Gerlach et al. 2013; Ghazanfar et al. 2016). Moreover, they are herbivorous insects, thus having direct contact to and feeding on treated vegetation, making them vulnerable to pesticide applications (Uhl and Brühl 2019). Direct effects of pesticides on butterflies have been in general rarely studied (Mulé et al. 2017; Braak et al. 2018), with the situation being even worse with regard to fungicides (but Olaya-Arenas et al. 2020; Riihimäki et al. 2025). Using controlled laboratory experiments, we here investigate to what extent the application of a difenoconazole fungicide to host plants interferes with the survival and development (development time and body size) of *P. napi* relative to untreated controls and a combination of fungicide and insecticide. We predict that individuals feeding on fungicide-treated plants will show reduced survival and detrimental sublethal effects compared to controls, while those feeding on plants treated with a combination of the fungicide and insecticide show very high mortality.

## Materials and methods

### Study organisms

The Green-Veined White, *Pieris nap*i L. (Lepidoptera: Pieridae), is a temperate-zone butterfly, being widely distributed across the Holarctic region including Europe, Asia, and North America (Chew and Watt 2006). It is a non-target species readily occurring in agricultural landscapes. *P. napi* thrives in a variety of habitats, though in Europe it prefers moist meadows and forest ecotones (Ebert and Rennwald 1991; Oliver et al. 2012). Depending on climate, the species has 1–3 generations a year; it overwinters in the pupal stage (Välimäki and Kaitala 2007; Peters et al. 2024). Females lay eggs singly on the undersides of the leaves of their host plants, viz. different species of the Brassicaceae family such as *Alliaria petiolata* and *Cardamine pratensis* (Friberg and Wiklund 2019). Females are polyandrous, and, accordingly, males are larger than females due to a positive correlation between body and spermatophore size, with larger spermatophores delaying female remating (Wiklund and Kaitala 1995).

### Study area, field sampling and oviposition

In May 2024, we collected 20 fecund *P. napi* females in the vicinity of Koblenz, Germany (50.3600, 7.5899). The climate around Koblenz is suboceanic with a mean temperature of 10.8 °C and a precipitation of 674 mm / year (1971–2000; klimadiagramme.de), enabling 2–3 generations of *P. napi* per year. Caught females were transferred to the University of Koblenz and kept in climate-controlled cabinets (Sanyo MIR-554-PE) with a photoperiod of 18 L:6 D, a constant temperature of 26 °C, and 70% relative humidity. They were housed individually in transparent 1-L plastic containers covered by gauze, which contained a fresh leaf of *Alliaria petiolata* for oviposition, water, and a 30 vol% sucrose solution. Resulting eggs were collected daily, transferred to glass vials, and kept at 22 °C. Before random allocation to treatments, hatchlings were fed fresh *A. petiolata* leaves for five days.

### Experimental design

Throughout, we used field-collected *A. petiolata* for larval feeding, being one of the main host plants of *P. napi* (Friberg and Wiklund 2019). Plants were collected along forest edges around Koblenz that did not border agricultural fields (minimum distance > 1 km). However, we cannot completely rule out that plants were exposed to some pesticides, but that would affect all treatments equally and thus not confound our comparisons. To investigate effects of fungicides and insecticides on the butterfly, we established the following treatments: control, one water spray dose (C1); control, two water spray doses (C2); fungicide, one spray dose (F1); fungicide, two spray doses (F2); fungicide and insecticide, one spray dose each (FI1); fungicide and insecticide, two spray doses each (FI2).

We used as fungicide “Compo Duaxo^®^” with the active compound difenoconazole (0.17 g/L), and as insecticide “Spruzit^®^ Pest-Free Rose Spray” containing pyrethrum (0.05 g/L) and rapeseed oil (8.25 g/L). Both products are designed for garden use and were applied as ready-to-use liquids. Note that, based on our design using formulations instead of active ingredients, we cannot determine with certainty whether difenoconazole per se or other ingredients of the fungicide cause potential lethal and sublethal effects (see also Discussion). Host-plant leaves were spread out on clean paper towels and then sprayed with one or two spray doses of (1) tap water for controls, (2) “Compo Duaxo^®^” for the fungicide treatments, and (3) “Compo Duaxo^®^” immediately followed by “Spruzit^®^” for the fungicide-insecticide treatments. The latter treatment was included as ‘negative control’, i.e. to estimate the effects of the fungicide relative to a mixture of fungicide and insecticide, which should, by definition, be lethal. For spraying, a hand-held plant sprayer with 1 L volume was used. For difenoconazole, 0.03 ± 0.001 mg per spray dose and 0.50 ± 0.02 µg per cm² were applied on average (means ± SE), for pyrethrum 0.02 ± 0.001 mg per spray dose and 0.36 ± 0.02 µg per cm² (Table A1, supplementary material). Treated leaves were air-dried for ca. two hours before being fed to the larvae.

On day 6 after hatching, larvae were randomly assigned to the above treatments, using 40 larvae each (total *n* = 240). All larvae were reared individually in 250 ml plastic containers lined with moist tissue paper and supplied with treated *A. petiolata* leaves. Containers were cleaned and supplied with fresh, treated food daily until pupation of the larvae. Larvae and resulting pupae were maintained in climate cabinets (Memmert HPP 260 ECO) at a 18 L:6 D photoperiod, light temperature of 25 °C, dark temperature of 18 °C, and a relative humidity of 75% to simulate natural environmental conditions (cf. Raharivololoniaina et al. 2021). Eclosed butterflies were frozen immediately after eclosion for later analyses. The following traits were scored for each individual: mortality, and for surviving individuals additionally larval and pupal development time (in days), pupal mass, larval growth rate (natural logarithm of pupal mass / larval time), thorax and abdomen mass, thorax-abdomen ratio, and wing area. To measure the latter traits, frozen butterflies were dissected by removing wings, heads, and legs. Then, thorax and abdomen were separated, weighed, and the thorax-abdomen ratio calculated. Forewing area was measured using digital images of right forewings. All masses were recorded using a microscale (Sartorius CPA225D).

### Statistical analyses

Variation in mortality rates across treatments were analyzed using chi-square tests (observed versus even) and Cox Proportional Hazards. The mean longevity of individuals (i.e. the time from egg hatching until death or pupation) across treatments was analyzed with a Kruskal Wallis ANOVA, followed by multiple comparisons of mean ranks using Dunn-Bonferroni tests. Due to high mortality (Table 1), only 65 individuals were available to investigate sublethal effects (69 out of 240 individuals survived, of which four had deformed wings and were therefore excluded from further analyses). Thus, we had to pool the control (C1 and C2; *n* = 53) and fungicide groups (F1 and F2; *n* = 11), while the single individual that had survived the fungicide-insecticide treatment was excluded from further analyses. Differences among both resulting groups were analyzed with Mann Whitney U tests. Additionally, we used general linear models (GLMs; with normal error distribution and log-link function) with treatment group and sex as fixed factors to account for variation among sexes. Statistical analyses were conducted using Statistica (12.0, Statsoft, Tulsa, USA).

**Table 1:** Overview over treatments, initial sample size, surviving individuals, survival in percent, and mean longevity of larvae until death or pupation in days

Treatment	Initial *n*	Survivors	Survival %	Longevity ± SD
C1	40	30	75.0	38.2 ± 12.4^a^
C2	40	26	65.0	35.0 ± 14.1^a^
F1	40	5	12.5	25.6 ± 12.1^ab^
F2	40	7	17.5	18.9 ± 14.3^b^
FI1	40	1	2.5	8.5 ± 7.9^c^
FI2	40	0	0.0	6.1 ± 1.7^c^

For the latter, different superscript letters indicate significant differences among groups (see Table A2, supplementary material). C1: control, one water spray dose, C2: control, two water spray doses, F1: fungicide, one spray dose, F2: fungicide, two spray doses, FI1: fungicide + insecticide, one spray dose, FI2: fungicide + insecticide, two spray doses

## Results

**Fig. 1:**
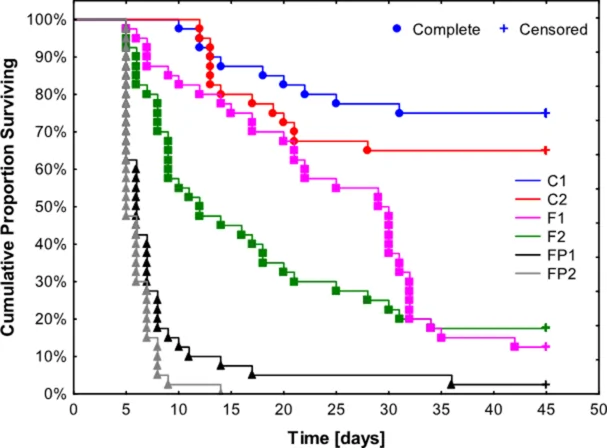
Cumulative survival probability of *Pieris napi* larvae across treatments. Survival rates were higher in control than in all other groups. C1: control, one water spray dose, C2: control, two water spray doses, F1: fungicide, one spray dose, F2: fungicide, two spray doses, FI1: fungicide + insecticide, one spray dose, FI2: fungicide + insecticide, two spray doses

Observed versus expected survival rates differed significantly across treatments (Chi^2^_5_ = 184.8, *p* < 0.0001), with survival being higher in the controls than in all other groups (Table 1). Similar results were obtained by a survival analysis (Cox Proportional Hazards; Chi^2^_5_ = 150.5, *p* < 0.0001), showing that larvae treated with a fungicide and an insecticide died very quickly, while those treated with a fungicide survived longer before eventually dying, which was particularly pronounced in the F1 treatment (Fig. 1). Accordingly, mean longevity was highest in control animals and lowest in fungicide-insecticide-treated ones (Table 1 and A2; supplementary material). Regarding other traits, control and fungicide-treated individuals differed significantly in larval time, pupal mass, larval growth rate, and wing area, with fungicide-treated individuals showing slower growth and being smaller than the control individuals (Table 2). GLMs with sex as factor revealed qualitatively identical results (Table A3, supplementary material). They additionally indicated significant sex differences in pupal mass (females: 170.1 ± 4.1; males: 189.9 ± 4.1), thorax-abdomen ratio (females: 0.343 ± 0.015; males: 0.404 ± 0.016), and a tendency in wing area (females: 224.4 ± 7.0; males: 241.4 ± 7.4).

**Table 2:** Results of Mann Whitney U tests for differences between control and fungicide-treated individuals of *Pieris napi*

Trait	Z	*p*	Control	Fungicide
Larval time [days]	-4.59	**< 0.0001**	9.76 ± 0.14	12.46 ± 0.31
Pupal time [days]	-0.13	0.9009	8.83 ± 0.09	8.91 ± 0.20
Pupal mass [mg]	2.30	**0.0217**	189.90 ± 2.95	168.56 ± 6.48
Growth rate [mg/day]	4.59	**< 0.0001**	0.542 ± 0.007	0.418 ± 0.016
Thorax mass [mg]	1.63	0.1035	11.29 ± 0.41	9.56 ± 0.90
Abdomen mass [mg]	0.99	0.3233	29.69 ± 0.84	27.27 ± 1.85
TA ratio	0.59	0.5571	0.383 ± 0.011	0.359 ± 0.024
Wing area [mm^2^]	2.88	**0.0039**	248.72 ± 4.87	215.76 ± 10.68

Given are test statistics and least square means ± standard error for each trait. Significant p-values are given in bold. TA ratio: thorax-abdomen ratio. n_control_ = 53, n_fungicide_ = 11

## Discussion

Our results on an insect herbivore, the butterfly *P. napi*, show that fungicide application to host-plant leaves reduced survival strongly compared to the control treatment, indicating the high toxicity of the fungicide investigated. The principle differences between fungicide and fungicide plus insecticide application were an even higher mortality in the latter, and that the former survived longer before eventually dying. This effect tended to be dose-dependent, as larvae exposed to two spray doses tended to die sooner (Table 1). Finding such high toxicity in a freely available, ready-to-use fungicide, which is only mildly harmful to insects according to the manufacturer, seems remarkable, as it can be used in gardens without any restrictions. Note that we applied ca. 0.5 µl / cm^2^ per spray dose (see above), which is considerably less than the dosage recommended for the difenoconazole fungicide “Syngenta Amistar Gold” (1.25 µl / cm^2^; www.baywa.de). Thus, our treatments involved field-realistic dosages, especially the F2 treatment, while the F1 treatment used a comparably low dosage still having strongly detrimental effects. Whether the high toxicity is caused by the active ingredient difenoconazole seems plausible (see further below) but cannot be unequivocally shown by our study because of using the entire formulation. Sublethal detrimental effects of the fungicide were also visible as prolonged development and reduced body size. In insects, fast development and large body size typically confer a higher fitness, based on a reduced risk of dying before reaching sexual maturity and an increased fecundity and stress resistance, respectively (Honěk 1993; Blanckenhorn 2000; Karl and Fischer 2008).

A major issue in toxicology studies is that typically active ingredients are tested in isolation rather than in combination with others, i.e. than entire formulations. This is despite the fact that the toxicity of commercial fungicides may be far higher than the sum of the toxicities of their active ingredients when assessed individually (Gomes et al. 2021; Jorge-Escudero et al. 2022). Consequently, for a realistic prediction of environmental hazards, the toxicity of formulated products rather than individual active ingredients must be explored (Gomes et al. 2021), as we have done here. With regard to fungicides, our current knowledge on their effects on non-fungal biodiversity suffers from limited research efforts, putatively based on the notion that they are less harmful than other pesticides, and from a neglect of insect herbivores in such studies (Zubrod et al. 2019; Sedlmeier et al. 2025; Wan et al. 2025; but Riihimäki et al. 2025).

Results very similar to ours were obtained in a recent study on another butterfly, *Melitea cinxia* (Riihimäki et al. 2025). Applying a fungicide with azoxystrobin and difenoconazole as active ingredients to the butterfly’s host plant substantially reduced survival, pupal mass and larval growth rates (Riihimäki et al. 2025). In the monarch butterfly, the fungicides azoxystrobin and trifloxystrobin reduced adult size significantly but survival only slightly (Olaya-Arenas et al. 2020). These findings may suggest that difenoconazole, or perhaps triazole fungicides in general, are particularly harmful to butterflies. Difenoconazole has been shown to also have negative effects on survival, morphology, detoxification, immunity, and neural pathways in honeybees (Kaur et al. 2023; Liu et al. 2025). Other triazole fungicides, namely tebuconazole and mefentrifluconazole, are toxic for e.g. the moth *Bombyx mori*, earthworms, fishes and rats (Yang et al. 2018; Li et al. 2019, 2020, 2022; Yao et al. 2024; Saeed et al. 2025).

Our study adds to the notion that fungicides are indeed an overlooked but potentially dangerous pesticide class (Zubrod et al. 2019). Evidence for toxic effects of fungicides on non-target organisms is currently accumulating at a fast rate (e.g., Marinho et al. 2020; Gomes et al. 2021; Jorge-Escudero et al. 2022; Riihimäki et al. 2025; Saeed et al. 2025). Pesticides may impose oxidative stress and suppress immune function in butterfly larvae, which may mechanistically underlie increased mortality and reduced performance (Sánchez-Bayo 2011; González-Santoyo and Córdoba-Aguilar 2012). Our results also indicate that investigating a few standard test organisms only while neglecting the large group of herbivorous insects, which are directly exposed to pesticides, as is currently the case EU insecticide registration protocol (Sedlmeier et al. 2025), is clearly insufficient to evaluate potential environmental hazards of pesticides. For instance, large variation among species in the sensitivity to fungicides, which may even occur within one taxon such as bees, has been shown (Jorge-Escudero et al. 2022; Jütte et al. 2023).

The sex differences found in our study, namely that males are the larger sex with a higher thorax-abdomen ratio, reflect the well-known biology of the species. Female protandry drives selection for large body size in males in *P. napi*, based on a positive correlation between body and spermatophore size with larger spermatophores delaying female remating (Wiklund et al. 1993; Wiklund and Kaitala 1995). A higher thorax-abdomen ratio in males than in females indicates a higher investment of males into flight ability and of females into reproduction, which is commonly found in Lepidoptera (Berwaerts et al. 2002; Teder and Tammaru 2005; Esperk et al. 2007).

## Conclusions

Overall, our results contribute to the growing body of evidence that fungicides, though often overlooked in insect risk assessments, can negatively affect insect survival and development. In particular difenoconazole fungicides seem to be highly problematic for butterflies and other insects, and we suspect that this may also apply to other triazole fungicides (see above). The current standards for the risk assessment of pesticides within the EU and elsewhere are evidently insufficient and should include herbivorous insects and also test entire formulations as well as interactive effects among different active ingredients in the future (see also Gomes et al. 2021; Jorge-Escudero et al. 2022; Sedlmeier et al. 2025). Based on the negative effects reported here and the widespread use of difenoconazole, it seems likely that fungicides are involved in the dramatic decline in (insect) biodiversity in agricultural landscapes (Raven and Wagner 2021; Mancini et al. 2023; Wan et al. 2025). Thus, there is an urgent need to overcome current deficiencies in regulations and to develop less harmful pesticides. 

## Electronic Supplementary Material

Below is the link to the electronic supplementary material.

supplementary material 1

## Data Availability

The datasets used during the current study are available from the corresponding author on request.
