# Supplementary material for: Toxicological effects of a difenoconazole fungicide on a non-target butterfly
Source: Ecotoxicology. 2026 Jul 31;35(6):149. doi: 10.1007/s10646-026-03133-5 (PMC13427870; doi:10.1007/s10646-026-03133-5)
Supplement: Supplementary file 1 — supplementary material 1 [file 10646_2026_3133_MOESM1_ESM.docx]

**Supplementary material**

**Table A1** Volume of the tested fungicide and insecticide per single spray dose (Δ mg) as well as active ingredients per spray dose and per cm^2^ of leaf. For measurements, one spray dose was applied into a petri dish with 10 replicates per pesticide. The volume per spray dose was assessed as mass gain per petri dish in milligrams. The amount of active ingredient per spray dose was then calculated as (mass gain / 1000) × concentration (g/L), using concentrations of 0.17 g/L for difenoconazole (fungicide) and 0.05 g/L for pyrethrum (insecticide). The amount per cm² was calculated by dividing the amount per petri dish by the petri dish area (63.62 cm²) and multiplying by 1000 to express values in µg/cm². For difenoconazole, 0.03 ± 0.001 mg per spray dose and 0.50 ± 0.02 µg per cm² were applied on average (means ± SE), for pyrethrum 0.02 ± 0.001 mg per spray dose and 0.36 ± 0.02 µg per cm².

**Table A1**

| **ID** | **Treatment** | **Initial Weight (mg)** | **Weight after spraying (mg)** | **Δ (mg)** | **Active ingredient per spray (mg)** | **Active ingredient per cm² (µg)** |
| --- | --- | --- | --- | --- | --- | --- |
| F1 | Fungicide | 40302.40 | 40481.40 | 179.00 | 0.03 | 0.48 |
| F2 | Fungicide | 52507.90 | 52673.60 | 165.70 | 0.03 | 0.44 |
| F3 | Fungicide | 53047.20 | 53243.30 | 196.10 | 0.03 | 0.52 |
| F4 | Fungicide | 53061.50 | 53267.90 | 206.40 | 0.04 | 0.55 |
| F5 | Fungicide | 47726.20 | 47915.10 | 188.90 | 0.03 | 0.50 |
| F6 | Fungicide | 54039.20 | 54212.00 | 172.80 | 0.03 | 0.46 |
| F7 | Fungicide | 50333.30 | 50496.50 | 163.20 | 0.03 | 0.44 |
| F8 | Fungicide | 51655.10 | 51845.80 | 190.70 | 0.03 | 0.51 |
| F9 | Fungicide | 52887.80 | 53071.60 | 183.80 | 0.03 | 0.49 |
| F10 | Fungicide | 54806.70 | 55032.60 | 225.90 | 0.04 | 0.60 |
| I1 | Insecticide | 47703.90 | 48216.00 | 512.10 | 0.03 | 0.40 |
| I2 | Insecticide | 48899.60 | 49374.00 | 474.40 | 0.02 | 0.37 |
| I3 | Insecticide | 47725.20 | 48164.90 | 439.70 | 0.02 | 0.35 |
| I4 | Insecticide | 52885.50 | 53360.50 | 475.00 | 0.02 | 0.37 |
| I5 | Insecticide | 46775.40 | 47131.60 | 356.20 | 0.02 | 0.28 |
| I6 | Insecticide | 53060.00 | 53424.40 | 364.40 | 0.02 | 0.29 |
| I7 | Insecticide | 50544.10 | 51005.20 | 461.10 | 0.02 | 0.36 |
| I8 | Insecticide | 51105.80 | 51638.30 | 532.50 | 0.03 | 0.42 |
| I9 | Insecticide | 52502.70 | 52981.80 | 479.10 | 0.02 | 0.38 |
| I10 | Insecticide | 51086.70 | 51568.00 | 481.30 | 0.02 | 0.38 |

**Table A2** Multiple comparisons of mean ranks for longevity based on Dunn-Bonferroni tests after Kruskal Wallis ANOVA. Significant p-values are given in bold.

|  | **C1** | **C2** | **F1** | **F2** | **FP1** | **FP2** |
| --- | --- | --- | --- | --- | --- | --- |
| **C1** |  | 1.0000 | 0.0642 | **0.0001** | **< 0.0001** | **< 0.0001** |
| **C2** | 1.0000 |  | 0.3512 | **0.0015** | **< 0.0001** | **< 0.0001** |
| **F1** | 0.0642 | 0.3512 |  | 1.0000 | **< 0.0000** | **< 0.0001** |
| **F2** | **0.0001** | **0.0015** | 1.0000 |  | **0.0034** | **< 0.0001** |
| **FP1** | **< 0.0001** | **< 0.0001** | **< 0.0001** | **0.0034** |  | 1.0000 |
| **FP2** | **< 0.0001** | **< 0.0001** | **< 0.0001** | **< 0.0001** | 1.0000 |  |

**Table A3** General linear model results for the effects of treatment group (control versus fungicide-treated) and sex on various traits in *Pieris napi*. Significant p-values are given in bold.

| **Larval time** | **DF** | **MS** | **F** | **p** |
| --- | --- | --- | --- | --- |
| Group | 1 | 77.43 | 66.22 | **< 0.0001** |
| Sex | 1 | 0.62 | 0.57 | 0.4521 |
| Error | 61 | 1.08 |  |  |
| **Pupal time** | **DF** | **MS** | **F** | **p** |
| Group | 1 | 0.053 | 0.12 | 0.7349 |
| Sex | 1 | 0.332 | 0.72 | 0.3988 |
| Error | 61 | 0.460 |  |  |
| **Pupal mass** | **DF** | **MS** | **F** | **p** |
| Group | 1 | 4016 | 10.94 | **0.0016** |
| Sex | 1 | 6246 | 17.00 | **0.0001** |
| Error | 61 | 367 |  |  |
| **Growth rate** | **DF** | **MS** | **F** | **p** |
| Group | 1 | 0.1388 | 52.93 | **< 0.0001** |
| Sex | 1 | 0.0048 | 1.83 | 0.1807 |
| Error | 61 | 0.0026 |  |  |
| **Thorax mass** | **DF** | **MS** | **F** | **p** |
| Group | 1 | 26.98 | 3.09 | 0.0837 |
| Sex | 1 | 18.93 | 2.17 | 0.1459 |
| Error | 61 | 8.73 |  |  |
| **Abdomen mass** | **DF** | **MS** | **F** | **p** |
| Group | 1 | 55.1 | 1.49 | 0.2276 |
| Sex | 1 | 75.2 | 2.03 | 0.1594 |
| Error | 61 | 37.1 |  |  |
| **TA ratio** | **DF** | **MS** | **F** | **p** |
| Group | 1 | 0.0047 | 0.85 | 0.3597 |
| Sex | 1 | 0.0593 | 10.71 | **0.0018** |
| Error | 61 | 0.0055 |  |  |
| **Wing area** | **DF** | **MS** | **F** | **p** |
| Group | 1 | 9724 | 8.10 | **0.0060** |
| Sex | 1 | 4619 | 3.85 | 0.0543 |
| Error | 61 | 1200 |  |  |
